# Cancer therapy disparity: unequal access to breast cancer therapeutics and drug funding in Canada

**DOI:** 10.3747/co.2007.153

**Published:** 2007-12

**Authors:** S. Verma, S. Sehdev, A.A. Joy

**Affiliations:** * Division of Medical Oncology, Toronto–Sunny-brook Regional Cancer Centre, Sunnybrook and Women’s College Health Sciences Centre, Toronto, Ontario; † The Oncology Group, William Osler Health Centre, Brampton, Ontario.; ‡ Cross Cancer Institute, Edmonton, Alberta

**Keywords:** Aromatase inhibitors, breast cancer, endocrine therapy

## Abstract

Adjuvant therapy has made a significant contribution in reducing breast cancer–specific mortality. Standard chemotherapeutics and tamoxifen have been the mainstay treatment for years, but recent clinical evidence supports the use of novel small-molecule therapy and aromatase inhibitor therapy in selected settings, challenging not only the traditional paradigm of breast cancer treatment, but also provincial funding of oncologic care across Canada. The disparity in access to aromatase inhibitor therapy for postmenopausal women with early-stage hormone-sensitive breast cancer across Canada is highlighted as an example.

## 1. INTRODUCTION

The aim of the *Canada Health Act* is to ensure that all eligible Canadian residents have reasonable access to publicly funded health care. The federal legislation requires comprehensive and universal access to publicly insured hospital, medical, and surgical–dental services uniformly, without any discrimination with regard to age, health status, or additional financial burden.

Innovative cancer therapies and advances in drug development have created new hope for patients and their health providers, but also contribute to an increase in health expenditures in an already cost-conscious environment^1^. A case in point is the aromatase inhibitors (ais), which are recommended as adjuvant therapy to lower the risk of tumour recurrence for postmenopausal women with early-stage hormone-dependent breast cancer. With cost-containment efforts being a major focus of all payers in Canada, widespread disparity in drug reimbursement exists among the provinces, cancer agencies, and hospitals.

In terms of the enormity of the health care issue, the statistics speak for themselves. One of every nine Canadian women is expected to develop breast cancer in her lifetime, and 1 of every 27 women is expected to die from breast cancer. As reported by the Canadian Cancer Society and the National Cancer Institute of Canada in *Canadian Cancer Statistics 2007,* an estimated 22,300 Canadian women will be diagnosed with breast cancer in 2007 and 5300 will die secondary to breast cancer (Table I), making this disease the most common cancer in women and the second leading cause of cancer mortality 2.

Mammographic screening and adjuvant therapies following breast cancer surgery have helped to contribute to a decline in annual mortality (1.2% per year since 1999) 2 ; however, despite a declining incidence of breast cancer in Canada in recent years, the current incidence of 104 cases per 100,000 (29% of all cancers) is still among the highest in the world according to the report, and the largest number of new cases occur in women between the ages of 50 and 59 years. In fact, breast cancer is the most common cancer in women under 50 years of age, in those 50 to 69 years of age, and in those 70 years of age and older, and it is the most common cause of cancer death in women under 50 years of age 2. In view of these high incidence and mortality rates, the need for prompt and early intervention with the most efficacious therapeutic regimen cannot be overstated 2.

Adjuvant therapy (local radiation or systemic treatment given after surgical resection for early-stage disease) for breast cancer was first used more than 100 years ago, but really moved forward following the discovery by Jensen and Jacobson of estrogen receptor action in the early 1960s 3. Approximately two thirds of postmenopausal breast cancer cases are estrogen-dependent 4–6, and through years of research, selective estrogen receptor modulators such as tamoxifen have become a cornerstone of treatment, reducing breast cancer recurrence and overall mortality in early-stage disease 7.

But treatment success has come at the cost of potential drug-related side effects—for example, the risk of thromboembolic disease and of endometrial cancers associated with the partial estrogen agonist effect of tamoxifen. Potent ais 8,9 were developed for complete estrogen blockade in postmenopausal women (by inhibiting the cytochrome P450 aromatase complex that converts peripheral androgens to estradiol), and compared with tamoxifen, they have demonstrated greater efficacy and a favourable side effect profile in both early- and late-stage hormone receptor–positive breast cancer 10–12.

Current key international guidelines support the use of these third-generation ais for the treatment of early breast cancer in postmenopausal women with hormone receptor–positive disease. The American Society of Clinical Oncology Technology Assessment Panel, the St. Gallen expert consensus, and the U.K. National Institute for Health and Clinical Excellence all recommend ais to lower the risk of tumour recurrence for postmenopausal women with early-stage hormone receptor–positive breast cancer 13–15. Strategies such as “upfront” ai therapy (substituting for 5 years’ tamoxifen therapy), or following tamoxifen therapy as an “early switch” (after 2–3 years of tamoxifen therapy) or as “extended adjuvant” therapy (after 5 years of adjuvant tamoxifen therapy) are currently supported.

## 2. EFFICACY OF UPFRONT AIs

Initial adjuvant therapy with an ai has been compared with tamoxifen in the Arimidex, Tamoxifen Alone, or in Combination (atac) trial (anastrozole) and in the Breast International Group (big) 1–98 (letrozole) trial 16–19.

In atac, disease-free survival was higher in the anas) trozole group at 33, 47, and 68 months (Table II 16–20. Benefits for the anastrozole group in terms of disease-free survival, time to recurrence, contralateral incidence, and time to distant recurrence persisted even after 5.7 years of follow up. Overall survival was similar between the two groups [hazard ratio (hr): 0.97; 95% confidence interval (ci): 0.85 to 1.12; *p* = 0.7] 16.

Similar results were obtained from an updated analysis of the big 1–98 monotherapy arms at a median follow-up of 51 months, confirming published evidence^19^ that letrozole monotherapy is superior to tamoxifen in the defined primary endpoint of disease-free survival (84.0% vs. 81.1%, *p* = 0.007), and in the secondary endpoint of time to distant recurrence (Table III; Coates AS. Letrozole versus tamoxifen: update of continuous therapy arms of big 1–98. Presented at the xxth Congress of the European Society for Medical Oncology; Istanbul, Turkey; September 29–October 3, 2006). At a median follow-up of 51 months, 352 disease-free survival events were seen among 2463 women receiving letrozole and 418 events among 2459 women receiving tamoxifen, reflecting an 18% reduction in the risk of an event (hr: 0.82; : ci 0.71 to 0.95; 95% *p* = 0.007) 21.

## 3. EFFICACY OF “SWITCH” AND “EXTENDED ADJUVANT” AI TRIALS

The results of the Intergroup Exemestane Study (ies) trial, the Austrian Breast and Colorectal Cancer Study Group (abcsg) 8 trial, the Arimidex–Novaldex (arno) trial, and the smaller Italian Tamoxifen Anastrozole (ita) trial all demonstrated the benefit of switching patients to an ai after 2–3 years of tamoxifen therapy. That switch significantly improved disease-free survival (local or metastatic recurrence, contralateral breast cancer, or death from any cause) as compared with standard adjuvant tamoxifen therapy (Table IV) 22–25. In addition, a modest improvement in overall survival was noted, with 222 deaths occurring in the exemestane group as compared with 261 deaths in the tamoxifen group (hr: 0.83; 95% ci: 0.69 to 1.00; *p* = 0.05) after 122 patients with estrogen receptor–negative disease had been excluded^26^.

Regardless of nodal status, extended adjuvant therapy may be warranted for patients completing about 5 years of tamoxifen therapy in view of continued risk of recurrence 27. In this regard, the final analysis of the results from the National Cancer Institute of Canada Clinical Trials Group ma.17 study confirmed that letrozole significantly reduces the risk of relapse, including distant metastases, as compared with placebo in women who remained disease-free for up to 3 months after completion of tamoxifen treatment. In addition, overall survival improved significantly in patients with node-positive disease at diagnosis 28.

## 4. SIDE EFFECTS OF AI THERAPY

In general, tamoxifen may have a role in patients with low risk of recurrence or poor tolerance for ais, but the use of third-generation ais is superior to tamoxi- fen in terms of certain toxicity profiles 29. The ais have a lower incidence of thromboembolic events and vaginal bleeding as compared with tamoxifen, and they do not increase the risk of endometrial neoplasia (and indeed may possibly be protective). Their impact on bone turnover and lipid metabolism varies with their individual safety profiles 30. Increased rates of arthralgia and myalgia are also seen with the ais.

## 5. THE CANADIAN SCENARIO

According to an online survey of 454 Canadian oncologists across 10 provinces, upfront adjuvant therapy with an ai is preferred for at least 50% of patients with postmenopausal estrogen receptor–positive early breast cancer 31. However, access to ai treatment strategies varies widely by province (Tables V, VI, VII).

### 5.1 Challenges with Reimbursement Strategies in Canada

Although the year 2006 saw a major breakthrough, with $260 million in federal funding for the Canadian Partnership Against Cancer 32, access to highly effective new drugs remains a major challenge for cancer patients. As health agencies review guidelines to define eligibility for treatment with ais, funding issues remain disparate and unresolved. Increasingly, the trend has been toward self-pay or third-party reimbursement by publicly funded cancer centres.

Currently, cancer reimbursement in Canada is managed provincially by agencies that create guidelines for drug usage aimed at standardizing patient care and cost expenditure, but also by provincial cancer programs and individual hospitals. A broad target patient population is the key rationale underlying drug reimbursement strategy, usually determined by a scientific process and review by disease-site groups. Provincially centralized care can offer great benefits if guidelines favour a new anticancer agent for incorporation into therapeutic regimens, but can also create a serious impediment for new treatments if delays occur in the writing of guidelines or in formulary listing.

In Quebec, for instance, where no standardized provincial clinical guidelines are in place, a Pharmacology Advisory Council recommends provincial formulary listing, but oncologists and pharmacists can drive the creation of formulary packages as needed. And although the Comité d’évolution des pratiques en oncology and the pgtm, a professional body of the four academic pharmacies (University of Quebec, University of Sherbrooke, McGill University, and the University of Montreal), offer drug advice and recommendations, individual hospitals make the final funding decisions. It is heartening to note, however, that ais are now fully approved and funded provincially. In Ontario, on the other hand, a Policy Advisory Committee provides Cancer Care Ontario with the recommended eligibility criteria for funding under the New Drug Funding Program after a comprehensive review of disease information with treatment recommendations, advanced clinical trial and pharmacoeconomic data, and manufacturer details. In fact, Ontario has extensive private pay options with individuals and their insurers footing the bill. On the other hand, the Atlantic provinces depend heavily on compassionate drug release by pharmaceutical manufacturers.

Indeed, the trend toward the private payment option for cancer drugs is increasing within the public health system in Saskatchewan, Ontario, New Brunswick, Nova Scotia, and British Columbia (a program in Alberta is ongoing). The financial risks and uncertainties involved in self-payment for expensive cancer drugs notwithstanding, third-party insurance plans and their inadequacies could create a major new challenge for the Canadian health care system.

Provinces such as Saskatchewan have no formulary listing for oncology products. Most private plans follow the Saskatchewan Prescription Drug Plan and therefore do not cover ais. However, full funding is available through the Saskatchewan Cancer Agency following requests from oncologists to the Provincial Oncology Drug Approval Committee on a case-by-case basis 33. In any case, as at December 2006, ais were still not approved for public funding. Access to ais is currently limited to private payers—self-pay, third-party insurer, or manufacturer’s compassionate release program—although the drugs are still administered by public cancer centres or hospitals (Table VII ) 34.

Oncologists in Manitoba follow the National Cancer Institute guidelines or other provincial (British Columbia or Ontario) recommendations. Cancer Care Manitoba (ccmb) launched the Clinical Practice Guidelines Initiative in January 2006 to develop evidence-based guidelines for local cancer care practice. The ccmb recommends funding based on a review of clinical data and outcomes by a multi-disciplinary tumour committee.^35^ Currently, ais are fully funded provincially in Manitoba (Table VII).

The Alberta Cancer Board’s Pharmacy and Therapeutics Committee considers oncologists’ requests to have a drug or treatment reviewed by a tumour group for development of provincial consensus guidelines and formulary listing under the Alberta Cancer Board Outpatient Cancer Drug Benefit Program. In a change over the 2005 status, the ais anastrozole, letrozole, and exemestane were approved for funding in 2006, with preferred agents to be used at selected time points in the course of therapy per the available clinical trial evidence (Table VII).

The British Columbia Cancer Agency (bcca) reimburses approved and indicated drugs for active cancer, without any ceiling on patient coverage, but within budgetary limits. Any new drug must be submitted to an appropriate bcca tumour group for approval and for further evaluation by various expert committees before it can be requested for inclusion under the bcca budget by the province’s Ministry of Health 36. However, payment in British Columbia for ais is based on prognostic factors. Upfront therapy with an ai is recommended for women with an elevated risk of early relapse, defined as high grade or low estrogen-receptor (1+) disease, or stage III disease (including any N2/N3, T4, or T3N+), and excluding women with low-grade T1N0 tumours. Sequential therapy is preferred for most postmenopausal women who are not at high risk for early relapse. Currently, all three of the ais are fully funded provincially for any of the time points in the adjuvant setting.

In Atlantic Canada outside of Nova Scotia, public funding of ais is restricted and extremely fragile, available only as second-line therapy for patients who have failed, are intolerant of, or have an absolute contraindication to tamoxifen. Submissions are considered based on health economic analysis and safety and efficacy data, although instances of compassionate release by the drug manufacturers are known. None of these provinces has any public funding program for ais. As in Ontario, patients in New Brunswick and Nova Scotia who are over 65 years of age or are under a social assistance program or private reimbursement can access ais. In Prince Edward Island, none of the ais has been funded. In Newfoundland and Labrador, access to ais is limited to patients 65 years of age and older, and to patients with disease-specific factors who are receiving social assistance and welfare (however, similar relative risk reductions have been shown for patients with breast cancer without these disease-specific factors).

### 5.2 Trends and Implications

In general, the disparity in funding and access to cancer drugs is quite remarkable across Canada and is not limited to ais alone. The western provinces, which have more integrated oncology drug budgets for both parenteral and oral drugs, have more uniform access to cancer drugs than do provinces with multiple drug-funding programs. The four western provinces of British Columbia, Alberta, Saskatchewan, and Manitoba have the fewest restrictions in terms of access to cancer drugs^34^. As at December 31, 2006, the four western provinces approved and funded 63 cancer drugs as compared with only 45 in the rest of Canada, which has possible implications for cancer-specific outcomes across the country. As an example, the provinces of New Brunswick, Nova Scotia, and Newfoundland and Labrador have limited access to the third-generation ais, and patients in Prince Edward Island have no access at all and depend entirely on compassionate release of the drugs by the manufacturer^34^.

## 6. CONCLUSION

In summary, provincial treatment guidelines with regard to ais vary widely (Table V), with Quebec and Prince Edward Island having no specific guidelines at all. Public-sector reimbursement in Canada for adjuvant ais therapy, unlike that for tamoxifen, is limited or restricted, although third-party insurance is available in most provinces. Table VII summarizes the accessibility to ai endocrine therapies and funding across Canada as at December 2006 34. A concordance between provinces in funding is needed, not only for ais as adjuvant therapy for breast cancer, but for all cancer therapies in general. Such a concordance will pave the way for the development of uniform national guidelines guaranteeing availability of prompt and efficacious therapeutic care for all cancer patients, wherever they reside in Canada.

In addition to evidence-based efficacy and safety analyses, cost is currently a critical factor in funding decisions relating to cancer therapeutics. Given these cost considerations, the need for transparent development of appropriate cost-effectiveness models to guide decision-making at all levels cannot be overstated. Government, pharmaceutical companies, third-party payers, self payers, and institutional sources all have joint responsibility for improving access to useful cancer therapeutics, and discussions at all levels should be undertaken in this regard. Although legislation such as Ontario’s *Transparent Drug System for Patients Act* 37 and formation of the Joint Oncology Drug Review panel are steps in the right direction, the effects with regard to increasing patient access to evidence-based therapy have yet to be felt.

## Figures and Tables

**TABLE I:** Estimates for female breast cancer in Canada, 2007^2^

Province	Age-standardized incidence rates (per 100,000)	New cases	Deaths	Age-standardized mortality rates (per 100,000)
Newfoundland and Labrador	101	370	100	27
Prince Edward Island	111	110	25	27
Nova Scotia	101	680	200	27
New Brunswick	100	540	130	23
Quebec	111	5900	1400	24
Ontario	104	8500	2000	23
Manitoba	107	810	210	25
Saskatchewan	98	630	150	22
Alberta	106	2000	440	22
British Columbia	93	2700	640	20

**TABLE II:** Disease-free survival in the Arimidex, a Tamoxifen, Alone or in Combination trial, patients with hormone receptor–positive tumours 20

Comparison	Follow-up								
	33 Months^17^			47 Months^18^			68 Months^16^		
	hr b	95% ci	*p* Value	hr b	95% ci	*p* Value	hr b	95% ci	*p* Value
Anastrozole vs. tamoxifen	**0.78**	0.65 to 0.93	0.003	**0.82**	0.70 to 0.96	0.014	**0.83**	0.73 to 0.94	0.005
Anastrozole vs. combination	**0.76**	0.63 to 0.91	0.002	nr			**Combination therapy discontinued**		

a AstraZeneca Canada, Mississauga, ON (generic name: anastrozole).

b Hazard ratios shown in bold type are statistically significant.

hr = hazard ratio; ci = confidence interval; nr = not reported.

**TABLE III:** Updated analysis of the Breast International Group (big) collaborative study 1-98 data

	Median follow-up			
	26 Months (big 2005 19)		51 Months (Coates 2006 a)	
	Letrozole	Tamoxifen	Letrozole	Tamoxifen
Patients (*N* )	4003	4007	2463	2459
Disease-free survival events	351	428	352	418
Systemic disease-free survival events	323	383	331	374
Deaths	166	192	194	211

a Coates AS. Letrozole versus tamoxifen: update of continuous therapy arms of big 1-98. Presented at the xxth Congress of the European Society for Medical Oncology; Istanbul, Turkey; September 29–October 3, 2006.

**TABLE IV:** Disease-free survival with aromatase inhibitors in sequential strategies

Trial	Treatment protocol	Follow-up (months)	hr	95% ci	*p* Value	Absolute risk reduction (%)	Years from randomization
ies 22, 23 (*n=*2362)	Switch to exemestane after 2–3 years	31	0.68	0.56 to 0.82	<0.001	4.7	3
abcsg-8/ arno^24^	Anastrozole treatment after 2 years	28	0.60	0.44 to 0.81	0.0009	3.1	3
ita 25	Switch to anastrozole at 2 years	36	0.35	0.18 to 0.68	0.001	5.8	3

ies = Intergroup Exemestane Study; abcsg-8/arno = Austrian Breast and Colorectal Cancer Study Group-8 trial and the Arimidex–Novaldex trials; ita = Italian Tamoxifen Anastrozole trial.

**TABLE V:** Public access to adjuvant endocrine therapy across Canada, as at July 2007

	Tamoxifen	Anastrozole	Letrozole	Exemestane
Ontario	Open	Limited use	Limited use	Limited use
Québec	Open	Open	Open	Open
Alberta	Open	Restricted	Restricted	Restricted
British Columbia	Open	Restricted (based on prognostic factors)	Restricted (based on prognostic factors)	Restricted (based on prognostic factors)
Saskatchewan	Open	Restricted	Restricted	Restricted
Manitoba	Open	Open (after deductible is met)	Open (after deductible is met)	Open (after deductible is met)
Atlantic Canada (Newfoundland and Labrador, Prince Edward Island, New Brunswick)	Open	Restricted	Restricted	Restricted
Nova Scotia	Open	Open	Open	Open

**TABLE VI:** Disparities in Canadian drug reimbursement guidelines for aromatase inhibitors (ais)

Quebec	No standard provincial guidelines Hospital pharmacy and therapeutics committees decide chemotherapy usage with formulary listing Régie de l’assurance maladie du Québec covers oral and intravenous chemotherapies Seniors 65 years of age and older have the choice of exclusive third-party insurance, if required, with open, unrestricted coverage for AIs and tamoxifen
Ontario	Cancer Care Ontario Clinical Practice Guideline or Evidence Summary used for reimbursement decisions on anticancer agents, reviewed by multidisciplinary Disease Site Groups ais are associated with some limited-use criteria in Ontario Drug Benefit program Ontario Drug Benefit covers seniors over 65 years of age, residents of long-term care facilities and Homes for Special Care, recipients of professional services under home care, and people on social assistance Trillium Drug Program covers those ineligible under Ontario Drug Benefit and lacking private insurance; requires patient deductibles and co-pays Third-party insurers also cover oral and hormonal therapies, supportive care outpatient treatments
Saskatchewan	Restricted public coverage of AIs
Manitoba	Manitoba’s Pharmacare Program reimburses AI therapies once patient deductibles are met
British Columbia	Public-sector reimbursement of AIs restricted, interchangeable, and based on prognostic factors Upfront therapy with an AI for women with an elevated risk of early relapse, defined as high grade or low estrogen receptor (1+) disease or stage III (including any N2/N3, T4, or T3N+) and excludes women with low-grade T1N0 tumours Sequential therapy is preferred for most postmenopausal women who are not at high risk for early relapse: Early switch to an AI for 2–3 years after 2–3 years of tamoxifen Late switch to an AI for 3 years after 3–5 years of tamoxifen, or if postmenopausal after 3 years of tamoxifen
Atlantic Canada	No specific guidelines on AI use Second-line AI therapy for patients who have failed, are intolerant of, or have an absolute contraindication to tamoxifen (with the exception of Nova Scotia)

**TABLE VII:** Aromatase inhibitor access and funding by drug and province, as at December 2006^34^

	Anastrozole (Arimidex a)	Letrozole (Femara b)	Exemestane (Aromasin c)
Access	C, S	C, S	C, S
British Columbia	A	A	A
Alberta	A	A	A
Saskatchewan	N, R, L4	N, R, L4	N, R, L4
Manitoba	A	A	A
Ontario	L2, L4	L2, L4	L2, L4
Quebec	A	A	A
New Brunswick	C, L2, L4	C, L2, L4	C, L2, L4
Prince Edward Island	N, C	N, C	N, C
Nova Scotia	L2, L4	L2, L4	N
Newfoundland and Labrador	C, L1, L2	C, L1, L2	C, L1, L2

a AstraZeneca Canada, Mississauga, ON.

b Novartis Pharmaceuticals Canada, Dorval, QC.

c Pfizer Canada, Kirkland, QC.

C = compassionate release from pharmaceutical company; S = self-pay or third-party insurer, drug readily available through retail pharmacies; A = approved and fully funded provincially; N = not approved or funded in that province; R = recommended for funding, but not yet approved, still pending; L1 = limited access on a case-to-case basis (disease-specific factors); L2 = limited access based on coverage for specific patient groups only (patients over 65 years of age, or those receiving social assistance or welfare); L4 = limited access based on private payment of the drug (self-pay, third-party insurer, or manufacturer’s compassionate program), but drug administered by public cancer centre or hospital.
